# Breast Cancer Screening for High-Risk Patients of Different Ages and Risk - Which Modality Is Most Effective?

**DOI:** 10.7759/cureus.945

**Published:** 2016-12-28

**Authors:** Elizabeth Wellings, Lauren Vassiliades, Reem Abdalla

**Affiliations:** 1 Medical Student, University of Central Florida College of Medicine; 2 Medical Student III, University of Central Florida College of Medicine

**Keywords:** mammography, mri, brca, survival impact, cost effectiveness, screening, specificity, sensitivity, high-risk patients, breast cancer survival

## Abstract

While the guidelines for breast cancer screening in average-risk women are well established, screening in high-risk women is not as clear. For women with BRCA1 or BRCA2 mutations, current guidelines recommend screening by clinical breast examination and mammography starting at age 30. For certain high-risk women, additional screening with magnetic resonance imaging (MRI) is encouraged. This review focuses on differentiating imaging modalities used for screening women at high-risk for breast cancer over the age of 50 by discussing the different imaging techniques, cost versus benefit, detection rates, and impact on survival. While mammography is the only imaging modality proven to reduce mortality from breast cancer, MRI is more sensitive in identifying cancers. MRI can often identify smaller malignancies at a greater resolution at an earlier stage. The use of MRI would be more cost effective as there would be less need for invasive therapeutic procedures. Research thus far has not identified an age-specific preference in imaging modality. There are no guidelines for high-risk women that specify screening with respect to age (i.e., older than 50 years old). More research is needed before screening guidelines in different age groups with various risk factors can be established.

## Introduction and background

Breast cancer is the most common cancer in women independent of race or ethnicity. In 2012, approximately 1.4 million invasive cancers were diagnosed. Of those diagnosed, 224,147 women (16%) and 2,125 men (0.15%) in the United States were diagnosed with breast cancer. In the same year, 582,607 cancer deaths were reported and of those deaths, 41,150 women (7%) and 405 men (0.07%) in the United States died from breast cancer [1]. Risk factors for breast cancer include age, genetic mutations (BRCA1 and BRCA2), younger age at menarche, first pregnancy after age 30, nulliparity, older age at menopause, physical inactivity, obesity, dense breast tissue, hormone replacement therapy, oral contraceptives, personal history of breast cancer or other breast diseases, family history of breast cancer, previous radiation therapy, and increased alcohol use [2].

For average-risk women, less than 15% lifetime risk of breast cancer using the Gail and Claus models, the United States Preventative Services Task Force (USPSTF) recommends that women who are 50 to 74 years old to get a mammogram every two years [3-4]. Mammography is the only imaging modality proven to reduce mortality from breast cancer. In high-risk women, magnetic resonance imaging (MRI) can be used along with mammography as a screening test. High-risk women include women with a known BRCA1 or BRCA2 mutation and their first-degree relatives, women with a lifetime risk of 20–25% or greater for breast cancer, and women with a history of chest irradiation between the ages of 10 and 30 [5]. Between 9,000 and 18,000 new diagnoses of breast cancer per year in the United States are associated with a genetic predisposition of which greater than 60% is due to a BRCA1 or BRCA2 mutation.

Management of women who are considered high-risk for the development of breast cancer is controversial, especially in women who carry a BRCA1 or BRCA2 mutation as they can develop cancers at an earlier age [6]. This is because mammography alone has its limitations in screening younger women with typically denser breast tissue or with specific tumor phenotypes [7]. For women with BRCA1 or BRCA2 mutations, current guidelines recommend to start annual MRI imaging at age 25 and to add mammography at age 30 [6]. The use of additional imaging, such as MRI, may be helpful in screening denser breast tissue [8]. However, there is not as much evidence supporting screening guidelines for high-risk women over the age of 50 whose breast tissue may not be as dense. In high-risk women over the age of 50, if a cancer or carcinoma in situ has not already been identified by any modality, there are no clear-cut guidelines as to how to continue screening these women. The American Cancer Society (ACS) recommends annual MRIs for high-risk women who remain healthy, i.e., whose life expectancy is estimated with at least five to seven years but does not specify guidelines with respect to increasing age [7]. Most practitioners will continue to screen women with annual MRI if they have a calculated increased risk. Risk is calculated every year to determine who still needs annual MRI using a risk model (e.g., the Claus model). Fortunately, in some women with age, breast tissue is known to become less dense, making it easier to identify lesions using mammography.

Our review focuses on differentiating imaging modalities used for screening women at high-risk for breast cancer over the age of 50 who have not already developed a malignancy, through a discussion of the different imaging techniques, cost versus benefit, detection rates, and impact on survival.

## Review

### Mammography versus MRI

For women over the age of 50 with a high-risk for breast cancer, such as having BRCA mutations, a family history of breast cancer, an early menstrual period, or late or no pregnancies, there is currently no uniform screening guidelines with respect to specific age and risk factors [2, 6]. According to the ACS, guidelines were updated in 2007 to reflect new evidence incorporating MRI as an adjunct to mammography for annual screening of high-risk women based on one or more of the following: BRCA mutation, a first-degree relative of BRCA carrier, or lifetime risk greater than or equal to 20-25%. Although this recommendation has been put in place, the age at which screening should begin and end for this population still has not been well established. Currently, there is no data supporting the effectiveness of screening high-risk patients with MRI in addition to mammography beyond the age of 69, as most current data is based on screening in younger high-risk women [9]. Organizations, such as the ACS and the USPSTF, have recommended different imaging strategies, each based on different high-risk patient populations. For example, the ACS recommends both MRI and mammography annually starting at the age of 30 for high-risk patients and to continue if the patient is still in good health while the USPSTF recommends high-risk patients to begin mammography in the 40's age bracket [3-4].

Although currently there is evidence and recommendations for the addition of MRI to mammography for younger high-risk patients, there has been insufficient evidence looking at the screening accuracy of patients over the age of 50. It is known that breast density tends to decrease with age, and the sensitivity of mammography increases as breast density decreases. In 2014, Phi. et al. performed a meta-analysis on individual patient data to evaluate whether there was a significant difference in sensitivity for patients less than 50 years compared to patients over 50 years. The results of the study showed that the sensitivity of MRI with mammography was significantly greater than mammography alone for patients over 50 years (P < 0.001). The study also showed that there was no significant difference in sensitivity when using MRI with mammography on patients less than 50 years old compared to patients over 50 years (P = 0.79). The study was based on six major high-risk screening trials (Table 1). The conclusions of this study state that MRI with mammography shows the same increase in sensitivity compared to mammography alone in older patients compared to younger high-risk patients, and therefore, limited screening with MRI should be considered for both age groups [10].

**Table 1:** Sensitivities and Specificities from Six Major International Studies Comparing MRI and Mammography in High-Risk Breast Cancer Patients

Location of the MRI Screening Study	United States [14]	Canada [13]	United Kingdom [12]	Netherlands [11]	Germany [8]	Italy [16]
MRI Sensitivity (95% CI)	100%	77%	77% (60-90)	80%	91%	94% (82-99)
Mammogram Sensitivity (95% CI)	33%	36%	40% (24-58)	33%	33%	59% (36-78)
MRI with Mammogram Sensitivity (95% CI)	N/A	N/A	94% (81-99)	N/A	93%	N/A
MRI Specificity (95% CI)	79%	95%	81% (80-83)	90%	97%	N/A
Mammogram Specificity (95% CI)	91%	>99%	93% (92-95)	95%	97%	N/A
MRI with Mammogram Specificity (95% CI)	N/A	N/A	77% (75-79)	N/A	96%	N/A

There is sufficient evidence based on randomized control trials (RCTs) and meta-analyses that mammography improves cancer mortality in the general population, but there is currently no evidence that it reduces mortality in high-risk breast cancer patients [5]. Therefore, in the 1990s, prospective studies were performed that evaluated the use of MRI as an adjunct screening modality to mammography specifically for these high-risk patients. The studies showed significantly higher sensitivities compared to mammography [5, 7-8, 11-16]. Sensitivities ranged from 71% - 100% with MRI alone versus 13% - 59% with mammography alone. In a meta-analysis performed on 11 studies, MRI alone showed a sensitivity of 77%, mammography alone showed a sensitivity of 39%, and mammography, plus MRI, showed a sensitivity of 94% [17].

Although MRI alone has shown to have high sensitivity for detecting breast cancers, it is limited by its lower specificity and thus increased false-positive rates. The increased false-positive rate increases the rate of recalls (call-backs for additional imaging) and biopsies [5, 9]. Although recall and biopsy rates have increased with the addition of MRI, it has been shown that with serial MRI studies, biopsy rates tend to decrease (Table 2). Relative to mammography, MRI has greater recall and biopsy rates but has greater detection rates and fewer false negatives [9].

**Table 2:** Recalls, Biopsies, and False-Negative Rates Resulting from MRI or Mammography Screening in the Netherlands Study Comparing MRI and Mammography in High-Risk Breast Cancer Patients [9, 11]

	MRI	Mammography
Recall rate	10.8%	5.4%
Biopsy rate	2.9%	1.3%
False-negative rate	0.2%	0.8%

While across breast cancer patients collectively, MRI and mammography combined have proven to be the most sensitive imaging modality for high-risk patients; different risk types may benefit from different imaging modalities. There are different types of high-risk patients, including BRCA mutations, radiation history, personal cancer history, history of lobular carcinoma in situ (LCIS), etc. Each risk type may benefit from a different surveillance program based on the type of breast cancer they are more likely to develop. For example, patients with a history of chest irradiation, such as Hodgkin lymphoma survivors, are at a high-risk for breast cancer; however, unlike BRCA patients, irradiated patients are more likely to have breast cancers with calcifications that are more easily found on mammography [5]. Furthermore, 13% - 20% of pediatric cancer patients treated with high-dose chest radiation will be diagnosed with breast cancer by the age of 40-45, and usually between the ages of 25-55 years, women’s breast tissue is quite dense; therefore, mammography alone is limited in this age group [18-22].

In patients with a personal history of breast cancer, post-treatment changes tend to distort the architecture and increase the density of the breast, therefore, limiting the effectiveness of mammography. In 2010, Brennan, et al., performed a retrospective study looking at 144 patients with a personal history of breast cancer. In the study, patients were screened with MRI, and 17 malignancies were detected. Ten of the 17 malignancies were mammographically occult, meaning it cannot be detected via mammogram. This study did not discuss the impact on survival but did state that the cancers detected via MRI were all minimal cancers (ductal carcinoma in situ or node-negative invasive cancer < 1 cm); therefore, it can be inferred that early detection of early stage cancer would decrease the mortality rate [23].

Regarding patients with a history of LCIS, there has been no consensus on the effectiveness of MRI in this patient group. While the ACS guidelines state that there is insufficient evidence for this patient group to recommend either for or against MRI screening, the National Comprehensive Cancer Network (NCCN) guidelines advise MRI as an adjunct to mammography. A study by Sung, et al. in 2013 have found that MRI has increased detection rates in LCIS patients up to 4.5% compared to mammography, and detected early T1 (tumor size ≤ 2 cm) invasive cancers that were found to be mammographically occult. Again, detection of early cancers with MRI indicate possible cure in these patients [24].

### Cost analysis

Cost-effectiveness of medical interventions and screening modalities can be quantified using several different parameters. For breast cancer, some parameters include cost per breast cancer detected, cost per breast cancer death averted, and cost per quality-adjusted life year (QALY) [25]. When discussing screening modalities, cost per year of life expectancy gained is most relevant. A study by Plevritis, et al. was performed on very high-risk women between ages 35 and 54 looking at the cost-effectiveness of mammography with MRI relative to mammography alone [26]. The study estimated that the cost per QALY to be $55,420 for BRCA1 patients and $130,695 for BRCA2 patients. When it came to patients with dense breasts, the estimates were $41,183 for BRCA1 and $98,454 for BRCA2 [26]. A study by Cott, et al. in 2012 also looked at the cost-effectiveness of dual-modality screening of MRI and mammography, also comparing its effectiveness for BRCA1 and BRCA2. The study concluded that alternating MRI and digital mammography at six-month intervals beginning at age 30, per current guidelines, was more cost-effective for patients with the BRCA1 mutation versus the BRCA2 mutation [27].

The Magnetic Resonance Imaging Breast Screening Study (MAR-IBS) looked at the cost effectiveness of moderately high-risk patients, those with a 50% likelihood of BRCA1 or BRCA2 (Table 3).

**Table 3:** Cost Analysis Comparing Mammography with MRI Relative to Mammography Alone

Cost per quality adjusted life year (QALY)	BRCA1	$55,420 [26]
	BRCA2	$130,695 [26]
	BRCA1 or BRCA2	$25,270 [28]
Cost per breast cancer detected	BRCA1 or BRCA2	$40,911 [12]

The study estimated the incremental cost of adding MRI to mammography to be $40,911 per cancer detected [12]. Taneja, et al. designed a model to evaluate the cost-effectiveness of screening high-risk women with MRI and mammography versus mammography alone. The model estimated the incremental cost per QALY for MRI and mammography versus mammography alone to be $25,270 compared to non-BRCA women whose estimates were found to be up to $315,210. The authors concluded that the MRI is cost-effective for high-risk patients [28]. The future technology of MRI aims to improve cost-effectiveness through reduction of exam time and elimination of the need for an IV contrast [29].

### Impact on survival

One of the major limitations of most studies that have compared MRI with mammography is that they have not been able to show evidence of decreased mortality. To date, only mammography has been validated as the imaging modality that has an impact on decreasing mortality.

There have been few studies that have looked at long-term follow-up of high-risk patients following surveillance with MRI. The Dutch MRI trial (30) found that four of the 42 mutations carriers diagnosed with invasive breast cancer died of the disease, and one had developed metastatic disease. The study reported an 84% distant disease-free survival at six years and an annual mortality rate of 1.2%. In the Passaperuma, et al. 2012 study with an eight-year follow-up, there was only one distant recurrence and death occurrence out of 54 mutation carriers diagnosed with breast cancer, which was equivalent to a 0.5% mortality rate for invasive cancers [31].

Surrogate markers for breast cancer mortality are currently tumor size and nodal status, which in addition to metastasis determine tumor stage [24]. Therefore, if MRI can detect cancers at an earlier stage, it can be inferred that there would be a decrease in mortality. Although one might be able to infer a decrease in mortality, more studies are needed to provide evidence of any decreased mortality rate. It is very unlikely that an RCT for MRI screening will take place; therefore, support for MRI in terms of decreased mortality will need to come from recurrence and survival data from observational studies.

## Conclusions

Mammography has long been the standard of preventative care for breast cancer screening in average-risk patients. When considering high-risk patients, research has identified the use of MRI as a more sensitive screening tool. Current guidelines are established for using MRI in younger high-risk patients but do not specifically clarify screening protocol for high-risk patients of older age and with different risk factors. MRI in combination with mammography has increased sensitivity in cancer detection for younger high-risk women. In addition, the use of MRI with mammography has proven to be the most cost-effective choice for high-risk women.

For older women, there has been insufficient evidence to support the use of MRI with mammography. This could be explained in part by decreased breast density with age allowing for equal detection rates with mammography alone. The most sensitive imaging modality may also vary with risk type depending on calcification, tissue architecture, and breast density. It is often unclear which imaging modality is the appropriate screening for high-risk women given the difference in age and risk factors. More research is needed to establish guidelines for screening high-risk women with regard to different ages and risk factors.
